# Supplementary material for: What, how and who: Cost-effectiveness analyses of COVID-19 vaccination to inform key policies in Nigeria
Source: PLOS Glob Public Health. 2023 Mar 22;3(3):e0001693. doi: 10.1371/journal.pgph.0001693 (PMC10032534; doi:10.1371/journal.pgph.0001693)

**S1 Appendix. Epidemiological model outline**

Model - before vaccine introduction


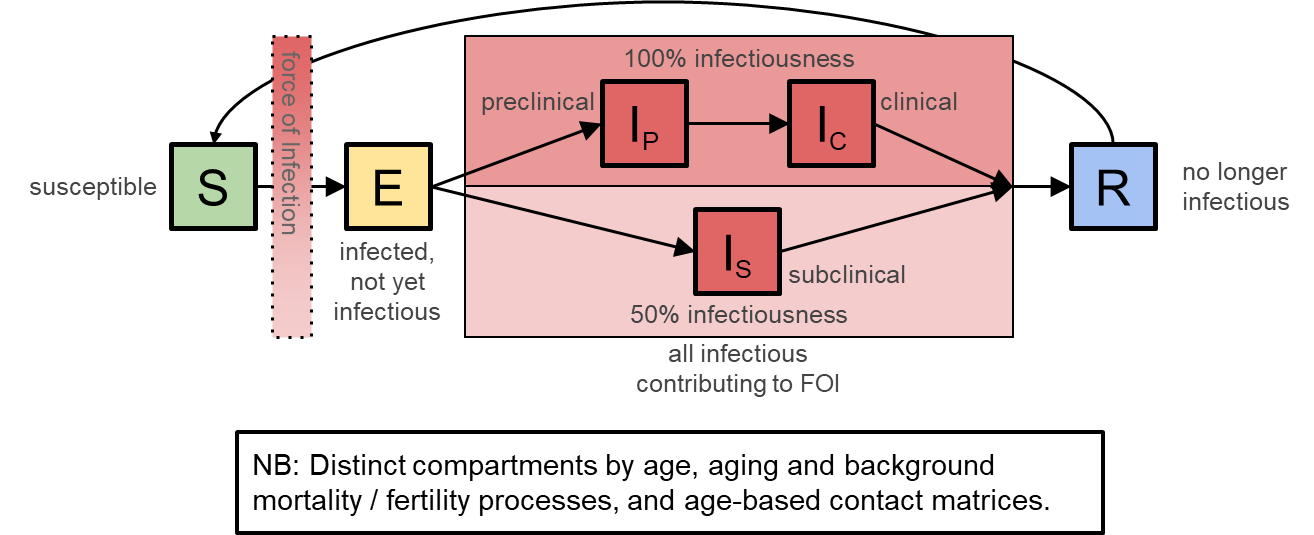


Model - introducing vaccine


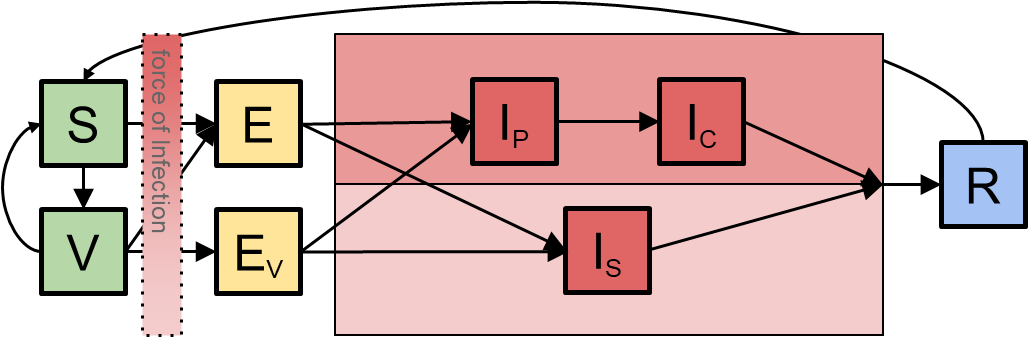

Supplement: S1 Appendix — (DOCX) [file pgph.0001693.s001.docx]
