# Supplementary material for: What, how and who: Cost-effectiveness analyses of COVID-19 vaccination to inform key policies in Nigeria
Source: PLOS Glob Public Health. 2023 Mar 22;3(3):e0001693. doi: 10.1371/journal.pgph.0001693 (PMC10032534; doi:10.1371/journal.pgph.0001693)
Supplement: S2 Appendix — (DOCX) [file pgph.0001693.s002.docx]

**S2 Appendix. Summary of the epidemiological parameters (including vaccine efficacy) used in the analyses**

| **Parameter** | **Base case value** | **Source** |
| --- | --- | --- |
| Latent period | Gamma (mean = 2.5, k=5) | Ferretti et al, 2020 [1]; Davies et al., 2021[2] |
| Contact rates | Age-dependent synthetic contact matrix for Nigeria | Prem et al, 2021 [3] |
| Proportion asymptomatic | Age-specific | Posterior from the CMMID COVID-19 Working Group [4] |
| Duration of infectiousness | Gamma (mean =5, k=4) | Ferretti et al, 2020 [1]; Davies et al., 2021 [2] |
| Duration of natural immunity | 1 year (average; exponentially distributed) | Assumed |
| Duration of vaccine immunity | 1 year (average; exponentially distributed) | Assumed |
| Vaccine efficacy/effectiveness | Viral vector 1: 75% (“AZ-like”) | Barnard RC, Davies NG, Jit M, Edmunds J, 2021. [5]  Table 1. “Pre-alpha / alpha” two dose estimates for the prevention of infection |
|  | Viral vector 2: 66% (“J&J-like”) | Bekker L-G, Garrett N, Goga A et al (2021). [6]  Prevention of hospitalisation as *upper* bound on the prevention of infection) |
|  | mRNA vaccines: 90% (“Moderna-like” and “Pfizer-BioNTech-like” vaccines) | Barnard RC, Davies NG, Jit M, Edmunds J, 2021. [5]  Table 1. Two dose estimates against disease |

**References**

1. Ferretti L, Ledda A, Wymant C, Zhao L, Ledda V, Abeler-Dörner L, et al. The timing of COVID-19 transmission. medRxiv. 2020:2020.09.04.20188516. doi: 10.1101/2020.09.04.20188516.

2. Davies NG, Barnard RC, Jarvis CI, Russell TW, Semple MG, Jit M, et al. Association of tiered restrictions and a second lockdown with COVID-19 deaths and hospital admissions in England: a modelling study. The Lancet Infectious Diseases. 2021;21(4):482-92. doi: 10.1016/S1473-3099(20)30984-1.

3. Prem K, Cook AR, Jit M. Projecting social contact matrices in 152 countries using contact surveys and demographic data. PLoS Comput Biol. 2017;13(9):e1005697. Epub 2017/09/13. doi: 10.1371/journal.pcbi.1005697. PubMed PMID: 28898249; PubMed Central PMCID: PMCPMC5609774.

4. Davies NG, Klepac P, Liu Y, Prem K, Jit M, Eggo RM. Age-dependent effects in the transmission and control of COVID-19 epidemics. Nat Med. 2020;26(8):1205-11. Epub 2020/06/18. doi: 10.1038/s41591-020-0962-9. PubMed PMID: 32546824.

5. Barnard R, Davies N, Jit M, Edmunds J. Autumn–winter scenarios 2021–2022. London School of Hygiene & Tropical Medicine, 2021.

6. Bekker L-G, Garrett N, Goga A, Fairall L, Reddy T, Yende-Zuma N, et al. Effectiveness of the Ad26.Cov2.S Vaccine in Health Care Workers in South Africa SSRN. 2021.
