# Supplementary material for: What, how and who: Cost-effectiveness analyses of COVID-19 vaccination to inform key policies in Nigeria
Source: PLOS Glob Public Health. 2023 Mar 22;3(3):e0001693. doi: 10.1371/journal.pgph.0001693 (PMC10032534; doi:10.1371/journal.pgph.0001693)
Supplement: S3 Appendix — (DOCX) [file pgph.0001693.s003.docx]

**S3 Appendix. Resource use assumptions**

| **Planning and coordination** | Each level of administration will require planning and coordination activities. All planning and coordination staff are assumed to be working with laptops. We assume staff salaries for:   - 20 senior government officials at the national level - 10 senior government officials per state (370 officials in total) - Seven junior government officials per local area (5,418 officials in total) - One team supervisor per facility (2,600 supervisors in total) |
| --- | --- |
| **Technical Assistance** | International consultants provide assistance on all aspects of vaccine rollout, from planning and coordination and social mobilisation to cold chain logistics and monitoring and evaluation. We assume all consultants are working with laptops and that teams of consultants require office space in each region. We assume salaries for:   - Four consultants for planning and coordination (including assistance with national trainings) - Two consultants for social mobilisation - Eight consultants for cold chain and logistics - Five consultants for monitoring and evaluation - Five consultants for service delivery |
| **Training** | Training is provided in the form of cascade training from national to subnational levels. Assuming bus transport for all participants of trainings, and hall rental, refreshments, and stationery for each training session.We assume staff salaries and per diems for:   - One national training of trainers (three days of training): Five senior level government officials and four international consultants conduct training for teams of junior level government officials and physicians from 37 states (three junior officials and three physicians per state) - One training per state (two days of training): conducted by three junior level government officials training teams of three nurses, two vaccinators, and two record keepers for each of the 774 local government areas - One training per local government area (two days of training): conducted by three nurses training teams of one vaccinator and one record keeper each per health facility |
| **Social mobilisation** | - One local consultant per state developing the social mobilisation strategy and messaging. - One local worker and one local leader per health facility to facilitate community awareness and events. - National TV ads: 60 second advertisement aired daily for six months - National radio ads: 60 second advertisement aired daily for six months - Flyers: 200 printed flyers per health facility - Local radio ads (at each local area): 60 second advertisement aired daily for one month |
| **Vaccine transport** | We assume full cold storage capacity at the national level for a single shipment of all doses, and we have allocated numbers of doses equally across regions. Assuming:   - Volume per vaccine dose : 3.76cm^3 - Refrigerated truck volume: 30,000L - Hilux double cab pickup volume (truck bed): 1,204L - Refrigerated truck vaccine storage capacity: 1,563 boxes per truck, 3,751,200 doses - Hilux truck vaccine storage capacity: five boxes per truck, 23,920 doses - Vaccine carrier volume (from woreda to facility): 2.7L, 717 doses - Fuel efficiency: 10km/L - Vaccines are picked up from local area refrigerators by motorcycle and delivered to facilities using long-range vaccine carriers (2.7L). - Three refrigerated trucks (and three drivers) at the national level - 1,659 hilux double cab trucks (and 1,659 drivers) at the local area and ward level - Average distance from state capital to Abuja: 515km - Average distance from local area capital to state capital: 109km - Average distance from health facility (or ward) to local area capital: 19km - Number of deliveries in country (to national stores): three - Number of deliveries per state by refrigerated truck: three (assuming four days for delivery) - Number of deliveries per local area by truck: seven (assuming two days for delivery) - Number of deliveries per ward by truck: three (assuming two days for delivery) - Number of deliveries per facility by motorcycle: 98 (assuming one day for delivery) |
| **Cold chain** | We assume office space rented at national and subnational level   - 15 30,000L cold rooms at the national level - One 10,000L cold room per state (37 in total) - One 145L main power fridges per state (37 in total) - One 92L solar direct drive fridge per local area (774 in total) - 0.5 50L solar direct drive fridge per ward (50% of wards have fridge - 4783 in total) - Each main power refrigerator uses 19,272 kilowatt-hours per year - Each solar direct drive refrigerator uses 12,176.4 kilowatt-hours per year - Each cold room uses 475,668 kilowatt-hours per year   Cold chain and logistics staff:   - National logistics working group: six members at national level and 37 at state level - State logistics working group: 10 members per state (370 in total) - Local area logistics working group: three members per local area (2322 in total) - Cold chain teams composed of one cold chain officer, one logistics officer, and one immunisation officer for six zones, 37 state, and 774 local government areas |
| **PPE** | Assuming only surgical masks and examination gloves used: three surgical masks per person per day and 10 pairs of gloves per vaccinator per day. |
| **Hand hygiene** | Hands washed before each dose: we assume 50% of handwashing is done with soap and water, and 50% of handwashing done with hand sanitiser. Facilities use water taps  1 litre of water used per hand wash, 1 mL of soap per hand wash. 3 ml of hand sanitiser per hand wash. |
| **Vaccine dose** | Assumptions:   - 15% wastage - 10% markup for freight cost (cost of delivering doses to country) |
| **Vaccine delivery** | - Doses delivered in facilities by three nurses, two record keepers per site per day - One syringe, one alcohol swab, plaster, and dry swab per dose - 1 minute spent handwashing, 5 minutes per dose - Two tables in facility-based delivery, five chairs for staff members plus 10 chairs for waiting area and for individual receiving vaccine |
| **Vaccination certificates** | One certificate per vaccinated individual, assuming 3 minutes for record keeping per dose.   - One FTE staff member per local area level entering data with a laptop into national vaccine database and office space |
| **Waste management** | - 5L safety box/sharps container: can contain 100 0.5ml syringes (20 syringes per nominal litre). - One biohazardous bag per delivery site per day for used PPE |
| **Pharmacovigilance** | Assuming all staff are working with laptops, and with office space per zone and per region. Assuming a rate of 12.98 serious adverse events per 100,000 doses, each serious AEFI would require 30 minutes of nurse time for management. We assume 15 chairs per vaccination site for vaccinated individuals to wait for 15 mins following immunisation.   - One record keeper for data entry, monitoring, and evaluation per state (37 in total) - One data manager overseeing 37 record keepers. - One AEFI kit per month |
