# Supplementary material for: What, how and who: Cost-effectiveness analyses of COVID-19 vaccination to inform key policies in Nigeria
Source: PLOS Glob Public Health. 2023 Mar 22;3(3):e0001693. doi: 10.1371/journal.pgph.0001693 (PMC10032534; doi:10.1371/journal.pgph.0001693)
Supplement: S4 Appendix — (DOCX) [file pgph.0001693.s004.docx]

**S4 Appendix. Costs, DALYs averted and ICERs per scenario**

**Scenario 1: Vaccination assuming 100% coverage for adults aged 50+ years old: analysis by vaccine type**

| **Vaccine type** | **Vaccine mechanism** | **Costs (2020 US$)** | **DALYs averted** | **ICER** |
| --- | --- | --- | --- | --- |
| Viral vector (AstraZeneca-like) | Disease | $17,173,361 | 105,792 | $162 |
| Viral vector (Johnson&Johnson-like) | Disease | $35,127,152 | 109,241 | $322 |
| mRNA (Pfizer-BioNTech-like) | Disease | $225,552,984 | 129,907 | $1,736 |
| mRNA (Moderna-like) | Disease | $399,527,682 | 129,907 | $3,075 |
| Viral vector (AstraZeneca-like) | Infection | -$31,997,592 | 194,811 | -$164 |
| Viral vector (Johnson&Johnson-like) | Infection | -$7,253,664 | 185,307 | -$39 |
| mRNA (Pfizer-BioNTech-like) | Infection | $160,851,899 | 239,802 | $671 |
| mRNA (Moderna-like) | Infection | $334,826,598 | 239,802 | $1,396 |

**Scenario 2: Vaccination assuming 90% coverage for all adults aged 18+ years old: analysis by vaccine type**

| **Vaccine type** | **Vaccine mechanism** | **Costs (2020 US$)** | **DALYs averted** | **ICER** |
| --- | --- | --- | --- | --- |
| Viral vector (AstraZeneca-like) | Disease | $240,016,554 | 419,026 | $573 |
| Viral vector (Johnson&Johnson-like) | Disease | $445,955,251 | 406,762 | $1,096 |
| mRNA (Pfizer-BioNTech-like) | Disease | $2,089,987,463 | 504,041 | $4,146 |
| mRNA (Moderna-like) | Disease | $3,558,231,256 | 504,041 | $7,059 |
| Viral vector (AstraZeneca-like) | Infection | $83,506,173 | 679,467 | $123 |
| Viral vector (Johnson&Johnson-like) | Infection | $248,527,565 | 726,496 | $342 |
| mRNA (Pfizer-BioNTech-like) | Infection | $1,960,288,906 | 706,408 | $2,775 |
| mRNA (Moderna-like) | Infection | $3,428,532,699 | 706,408 | $4,853 |

**Scenario 3: Vaccination assuming 100% coverage of all adults aged 50+ years old: analysis by delivery method**

| **Vaccine type** | **Delivery method** | **Vaccine mechanism** | **Costs (2020 US$)** | **DALYs averted** | **ICER** |
| --- | --- | --- | --- | --- | --- |
| Viral vector (AstraZeneca-like) | Health facility | Disease | $12,382,588 | 105,792 | $117 |
| Viral vector (AstraZeneca-like) | Campaign | Disease | $21,943,350 | 105,792 | $207 |
| Viral vector (AstraZeneca-like) | Targeted campaign | Disease | $22,047,271 | 105,792 | $208 |
| mRNA (Moderna-like) | Health facility | Disease | $394,736,909 | 129,907 | $3,039 |
| mRNA (Moderna-like) | Campaign | Disease | $404,297,671 | 129,907 | $3,112 |
| mRNA (Moderna-like) | Targeted campaign | Disease | $825,365,186 | 129,907 | $6,353 |
| Viral vector (AstraZeneca-like) | Health facility | Infection | -$36,788,365 | 194,811 | -$189 |
| Viral vector (AstraZeneca-like) | Campaign | Infection | -$27,227,603 | 194,811 | -$140 |
| Viral vector (AstraZeneca-like) | Targeted campaign | Infection | -$27,123,681 | 194,811 | -$139 |
| mRNA (Moderna-like) | Health facility | Infection | $330,035,824 | 239,802 | $1,376 |
| mRNA (Moderna-like) | Campaign | Infection | $339,596,587 | 239,802 | $1,416 |
| mRNA (Moderna-like) | Targeted campaign | Infection | $339,700,508 | 239,802 | $1,417 |

**Scenario 4: Vaccination assuming 25% coverage of all adults first prioritising all 50+ year olds: analysis by delivery method**

| **Vaccine type** | **Delivery method** | **Vaccine mechanism** | **Costs (2020 US$)** | **DALYs averted** | **ICER** |
| --- | --- | --- | --- | --- | --- |
| Viral vector (AstraZeneca-like) | Health facility | Disease | $38,026,353 | 219,359 | $173 |
| Viral vector (AstraZeneca-like) | Campaign | Disease | $60,878,282 | 219,359 | $278 |
| Viral vector (AstraZeneca-like) | Targeted campaign | Disease | $59,895,403 | 219,359 | $273 |
| mRNA (Moderna-like) | Health facility | Disease | $946,146,332 | 267,707 | $3,534 |
| mRNA (Moderna-like) | Campaign | Disease | $968,752,540 | 267,707 | $3,619 |
| mRNA (Moderna-like) | Targeted campaign | Disease | $968,015,381 | 267,707 | $3,616 |
| Viral vector (AstraZeneca-like) | Health facility | Infection | $-88,210,250 | 429,601 | -$205 |
| Viral vector (AstraZeneca-like) | Campaign | Infection | $-65,358,322 | 429,601 | -$152 |
| Viral vector (AstraZeneca-like) | Targeted campaign | Infection | $-66,341,201 | 429,601 | -$154 |
| mRNA (Moderna-like) | Health facility | Infection | $803,496,136 | 504,283 | $1,593 |
| mRNA (Moderna-like) | Campaign | Infection | $826,102,345 | 504,283 | $1,638 |
| mRNA (Moderna-like) | Targeted campaign | Infection | $825,365,186 | 504,283 | $1,637 |

**Scenario 5: Vaccination with a viral vector vaccine (AstraZeneca-like): analysis by age prioritization and target coverage**

| **Age prioritisation and target coverage** | **Vaccine mechanism** | **Costs (2020 US$)** | **DALYs averted** | **ICER** |
| --- | --- | --- | --- | --- |
| 25% adults (prior. 50+) | Disease | $49,354,030 | 219,359 | $225 |
| 100% 50+ | Disease | $17,173,361 | 105,792 | $162 |
| 90% adults | Disease | $240,016,554 | 419,026 | $573 |
| 70% 50+ | Disease | $36,933,745 | 142,580 | $259 |
| 70% 50+, 25% 18-49 | Disease | $87,248,888 | 226,197 | $386 |
| 25% adults (prior. 50+) | Infection | -$76,882,574 | 429,601 | -$179 |
| 100% 50+ | Infection | -$31,997,592 | 194,811 | -$164 |
| 90% adults | Infection | $83,506,173 | 679,467 | $123 |
| 70% 50+ | Infection | -$33,362,130 | 268,114 | -$124 |
| 70% 50+, 25% 18-49 | Infection | -$91,000,472 | 526,873 | -$173 |

**Scenario 6: Vaccination with an mRNA vaccine (Moderna-like): analysis by age prioritization and target coverage**

| **Age prioritisation and target coverage** | **Vaccine mechanism** | **Costs (2020 US$)** | **DALYs averted** | **ICER** |
| --- | --- | --- | --- | --- |
| 25% adults (prior. 50+) | Disease | $957,375,720 | 267,707 | $3,576 |
| 100% 50+ | Disease | $399,527,682 | 129,907 | $3,075 |
| 90% adults | Disease | $3,558,231,256 | 504,041 | $7,059 |
| 70% 50+ | Disease | $329,989,975 | 176,020 | $1,875 |
| 70% 50+, 25% 18-49 | Disease | $1,352,639,860 | 276,397 | $4,894 |
| 25% adults (prior. 50+) | Infection | $814,725,525 | 504,283 | $1,616 |
| 100% 50+ | Infection | $334,826,598 | 239,802 | $1,396 |
| 90% adults | Infection | $3,428,532,699 | 706,408 | $4,853 |
| 70% 50+ | Infection | $241,494,145 | 333,941 | $723 |
| 70% 50+, 25% 18-49 | Infection | $591,580,561 | 595,512 | $993 |
