# Supplementary material for: What, how and who: Cost-effectiveness analyses of COVID-19 vaccination to inform key policies in Nigeria
Source: PLOS Glob Public Health. 2023 Mar 22;3(3):e0001693. doi: 10.1371/journal.pgph.0001693 (PMC10032534; doi:10.1371/journal.pgph.0001693)
Supplement: S5 Appendix — (DOCX) [file pgph.0001693.s005.docx]

**S5 Appendix.** **Total vaccination and COVID-19 management costs over 5 years**

Scenario: 25% of adults (but first prioritising all those aged 50 years and above (Health facility)

Viral vector (AZ-like)

mRNA (Moderna-like)
