# Supplementary material for: What, how and who: Cost-effectiveness analyses of COVID-19 vaccination to inform key policies in Nigeria
Source: PLOS Glob Public Health. 2023 Mar 22;3(3):e0001693. doi: 10.1371/journal.pgph.0001693 (PMC10032534; doi:10.1371/journal.pgph.0001693)
Supplement: Attachment — Submitted filename: Author Responses Nigeria Paper.docx [file pgph.0001693.s006.docx]

We are grateful to the reviewers for the considered and valuable comments.

Below are our responses.

**Francis Ruiz and Sergio Torres-Rueda**

**Reviewer 1**

| **Reviewer’s Comments** | **Authors’ Responses** |
| --- | --- |
| Ethics statement: An ethics statement is a one-page essay that gives a picture of your core values and what potential supervisors, colleagues, or clients can expect from working with you. These must be clearly stated or mentioned.  Ethical considerations in research are a set of principles that guide your research designs and practices. These principles include voluntary participation, informed consent, anonymity, confidentiality, potential for harm, and results communication.So provide the ethical considerations in this research | We thank the reviewer for emphasising the importance of ethical considerations in research. We share his/her concerns. We have added a more extensive ethics statement elaborating our commitment to openness and transparency, as well as stating that the work is original, and reflects the work carried out by authors (all of whom take responsibility for the information published). Further, we clarify that, given that all data used in the work is publicly available, there was no need to obtain informed consent nor develop protocols on data anonymisation. Please see page 6. |
| Objective: The objective of this study was not clearly stated | We have removed the last sentence of the introduction and introduced a new statement under ‘Study design’ that says the following: “The objective of this study is to evaluate the cost-effectiveness of COVID-19 vaccination in Nigeria taking into account different vaccine alternatives, delivery platforms, and age groups.” (Page 7) |
| Discussion  Second statement: Taking a health system perspective only would suggest that viral vector vaccines (similar to those produced by AstraZeneca and Johnson & Johnson) may represent more cost-effective choices from the Nigerian perspective.  ‘’Only’’ should be changed to ‘’one’’ | We have sought to clarify the statement to read as follows: “Our analysis suggests that viral vector vaccines (similar to those produced by AstraZeneca and Johnson & Johnson) may represent more cost-effective choices from the Nigerian health system perspective.”. This is found on page 19. |
| Similar findings are seen for all the vaccines– this statement needs to be referenced. | To clarify the statement we have reworded it as follows:”Similar findings were seen for all the vaccines in our analysis in terms of how the costs were distributed over the 5-year period, although overall magnitudes differed”. (page 20) |
| Conclusion  The mode of delivery of vaccines has little effect on the cost effectiveness of the vaccine options. Provide the analysis/table that proves or supports this statement. | This comment in the conclusion is referencing the findings presented earlier in the Results section which looked at the question of how vaccines should be delivered (Figures 3 and 4). We have now replaced “mode” with “method” to ensure consistency with the text earlier in the paper (see page 24). |
| References  Vancouver reference style was used in the text while APA style was used in the list of reference. This is a contradiction. In Vancouver reference style, et al comes after listing 6 authors and their initials. All references in the list of reference must be re-written to conform with Vancouver reference style | We have ensured that the reference format is consistent and meets the journal requirements. |
| Implications of the study: This should be properly stated | We have added additional statements to the discussion that notes the following: “The findings from our study could be extrapolated to other settings; countries purchasing vaccinations against COVID-19 should ensure value for money when choosing among alternatives. As is the case in similar studies based in LMICs, we find that price per dose will probably be an important determinant of cost-effectiveness, as well as the group prioritised for vaccination (2),(29). How vaccines are delivered to eligible populations should be based on the most feasible arrangements available in the country as the cost-effectiveness of different delivery methods is similar. Finally, this work shows the viability, and indeed desirability, of developing combined epidemiological and economic models in these settings, although further work is needed in aligning methods when, for example, considering equity (36).”  (pages 23- 24) |
| Contribution to body of knowledge: The study is so germane for contribution to knowledge to be omitted. | We have commented on the existing evidence on cost-effectiveness of the COVID-19 vaccine (see pages 19-20). We have situated our results within the body of knowledge on the subject. We have pointed out that, as with other published economic evaluations (including Pakistan and Kenya), the price of the vaccine is a key driver of cost-effectiveness, as is the age prioritisation.  We have also highlighted in the conclusion how this work shows the viability of developing combined epidemiological and economics modelled in these settings (page 24) (See also response above). |

**Reviewer 2**

| **Reviewer’s Comments** | **Authors’ Responses** |
| --- | --- |
| Language; simple and clear, no grammatical errors  Flow; coherent and logical flow of information in all sections.  Literature review; extensive and relevant  Methods, Data analysis; detailed, focused, contains all the necessary information. The choice of models was relevant to the topic  Results and discussion; all important elements as guided by analysis frameworks were presented, and discussed.  Conclusions; in line with the main results  Usefulness of the study; very relevant to the contexts of LMIC | We thank Reviewer 2 for their encouraging comments on our work. |
